# Sexual development dysgenesis in interspecific hybrids of Medaka fish

**DOI:** 10.1038/s41598-022-09314-6

**Published:** 2022-03-30

**Authors:** A. L. Martinez-Bengochea, S. Kneitz, A. Herpin, R. H. Nóbrega, M. C. Adolfi, M. Schartl

**Affiliations:** 1 Department of Developmental Biochemistry, Biocenter, University of Wuerzburg, Am Hubland, 97074 Wuerzburg, Germany; 2 Reproductive and Molecular Biology Group, Department of Structural and Functional Biology, Institute of Biosciences, UNESP, Botucatu, Brazil; 3 Biochemistry and Cell Biology, Biocenter, University of Wuerzburg, Am Hubland, 97074 Wuerzburg, Germany; 4 UR 1037 Fish Physiology and Genomics, INRAE, 35000 Rennes, France; 5 Department of Chemistry and Biochemistry, The Xiphophorus Genetic Stock Center, Texas State University, San Marcos, TX 78666 USA

**Keywords:** Developmental biology, Evolution, Genetics, Molecular biology

## Abstract

Fish are amongst vertebrates the group with the highest diversity of known sex-determining genes. Particularly, the genus *Oryzias* is a suitable taxon to understand how different sex determination genetic networks evolved in closely related species. Two closely related species, *O. latipes* and *O. curvinotus,* do not only share the same XX/XY sex chromosome system, but also the same male sex-determining gene, *dmrt1bY*. We performed whole mRNA transcriptomes and morphology analyses of the gonads of hybrids resulting from reciprocal crosses between *O. latipes* and *O. curvinotus*. XY male hybrids, presenting meiotic arrest and no production of sperm were sterile, and about 30% of the XY hybrids underwent male-to-female sex reversal. Both XX and XY hybrid females exhibited reduced fertility and developed ovotestis while aging. Transcriptome data showed that male-related genes are upregulated in the XX and XY female hybrids. The transcriptomes of both types of female and of the male gonads are characterized by upregulation of meiosis and germ cell differentiation genes. Differences in the parental species in the downstream pathways of sexual development could explain sex reversal, sterility, and the development of intersex gonads in the hybrids. We hypothesize that male-to-female sex reversal may be connected to a different development time between species at which *dmrt1bY* expression starts. Our results provide molecular clues for the proximate mechanisms of hybrid incompatibility and Haldane’s rule.

## Introduction

Sex determination defines the fate of the bipotential gonad in an organism. After the developmental decision is taken the following period of sex differentiation establishes the functional cell types of the ovary or testis^1,2^. Among vertebrates, fish is probably the most diverse group with respect to sex-determination mechanisms and sex chromosome systems^3^, which can differ even between closely related species^3,4^. This extraordinary diversity of sex-determining mechanisms between related species is thought to have arisen by the addition, modification, or replacement of regulators at the upstream end of a common sex-determining pathway^5^. Remarkably, despite the variability in the genetic regulatory networks between different species that involved different sex-determining genes and sex differentiation pathways often employing opposing genetic mechanism or totally unrelated molecular players, the morphological, cell biological and functional outcome of the process remains constant, namely either ovary or testis^2,6^.

Sex determination variability is well studied within the genus *Oryzias*, commonly known as ricefishes^7^. Species of this genus have different sex chromosome systems and sex determining genes^7,8^. To date, the sex determining genes are known for four species of *Oryzias*: *O. latipes* (*dmrt1bY* or *dmY*), *O. curvinotus* (*dmrt1bY* or *dmY*), *O. luzonensis* (*gsdfY*), and *O. dancena* (*sox3Y*)^7^. Both, *O. latipes* and *O. curvinotus* have a XX/XY chromosome system and a karyotype of 2N = 48 chromosomes^9,10^. In both species, gonadal sex differentiation is triggered by the same master sex determining gene *dmrt1bY* (*dmY*), which is linked to the Y-chromosome^11–15^ and expressed at stage 36–37 (6–7 days after fertilization) in *O. latipes* male embryos^15^. Several studies have shown that *dmrt1bY* is necessary and sufficient for triggering testicular differentiation^16–18^. This gene arose after a duplication/insertion event of the autosomal *dmrt1* gene around 10 million years ago (MYA) in a common ancestor of both species^18^. Belonging to the Doublesex and Mab-3 or DM protein family, the Dmrt1bY protein has a DNA-binding domain and acts as transcription factor^6^. During gonadal sex differentiation of *O. latipes*, the first sign of morphological differentiation is an increase of germ cell numbers and first meiosis in females at stage 38, while these processes occur much later in males (around 8 days after hatching)^15^.

As such ricefish provide a unique system for conducting cellular and molecular analyses of interspecific hybrids in order to better understand the phenomena described as hybrid incompatibility and hybrid vigor in relation to sexual development. In addition, natural hybridization between species of the Oryzias genus was already reported^19^. Since *O. latipes* and *O.curvinotus* do not only share the same chromosome system, but also, the same number of chromosomes and same sex determining gene (*dmrt1bY*), it is tempting to assume that the regulatory pathways involved in sexual development are conserved between both species. However, earlier studies demonstrated that interspecific hybrids between *O. latipes* and *O. curvinotus* are viable but were compromised to—various extents—with respect to fertility^20,21^. In addition, female hybrids produce diploid eggs by premeiotic endomitosis, which can generate polyploid individuals after regular crosses with fertile males^22,23^. Into that perspective hybrids between both species can give important information about how their respective molecular pathways work and influence each other in order to regulate and control sex determination and gonad differentiation in different genetic backgrounds. To understand the molecular mechanisms underlying hybrid sterility and male-to-female sex reversal in the interspecific crosses of these two sister species sharing the same sex determining gene, morphological and molecular analyses were performed on F1 hybrids between *O. latipes* females and *O. curvinotus* males and from the reciprocal cross. We analyzed gonadal development in embryos and adults of parental fish as well as in their hybrid progeny. As results, morphological analyses of the gonads showed an overabundance of germ cells and ovotestis in aging female hybrids. Unexpectedly transcriptome analyses of adult gonads of parental fish revealed that, the expression profiles of the genes involved during gonadal development are not conserved between both species. Remarkably, ovaries of XY sex-reversed fish are morphologically and molecularly similar to the XX hybrid gonads.

The results emphasize the extreme plasticity of the gene regulatory networks underlying gonadal determination and formation even in very closely related species sharing the same master sex determiner factor and provide a molecular link to the hybrid incompatibility and Haldane’s rule.

## Materials and methods

### Ethics statement and the ARRIVE guidelines

All animals were kept and sampled in accordance with the applicable EU and national German legislation governing animal experimentation. In particular all experimental protocols were approved through an authorization (55.2532-2-215) of the Veterinary Office of the District Government of Lower Franconia, Germany, in accordance with the German Animal Protection Law (TierSchG) and in accordance with ARRIVE guidelines.

### Animals

*Oryzias latipes* and *Oryzias curvinotus* as well as hybrid fish were raised in the fish facilities of the Biocenter of the University of Würzburg. For detailed description of this model species and its features see^24^. All experiments were performed using the Carbio strain, *O. latipes*. Founder fish for the *O. curvinotus* strain were obtained from NBRP Medaka (https://shigen.nig.ac.jp/medaka/). All animals were kept under standard photoperiod cycle of 14 h/10 h light/dark at 26 °C (± 1 °C). Fertilized eggs were collected 1–2 h after starting the light cycle and embryos raised at 26 °C in Danieau’s medium (17.4 mM NaCl, 0.21 mM KCl, 0.12 mM MgSO_4_, 0.18 mM Ca(NO_3_)_2_, 1.5 mM Hepes, pH 7.2). The stages of development were assigned according to Iwamatsu (2004).

Hybrids were obtained by crosses and reciprocal crosses of *O. latipes* and *O. curvinotus* in single pair matings. Hybrids were named H:Y^*lat*^ when the male was from *O. latipes*, and H:Y^*cur*^ when the male originated from *O. curvinotus,*. Phenotypes and histological analysis were performed on fish at 1 or 2 years of age.

### Genotyping of embryos and adult fish

The phenotypic sex of adult fish was established after analysis of their secondary sexual characters, namely the shape of the dorsal and anal fins, and papillary processes on the male anal fin rays. Sex phenotyping was also performed by gonad histological analysis. The gonads of medaka fish are fused during early stages of development, which are present at the adult animals as a single organ after dissection.

To determine the genotypic sex of embryos and adult fish (XY or XX), genomic DNA was extracted. For *O. latipes* and Hybrids (H:Y^*lat*^), caudal fin clips of adult fish or the whole dissected head of embryos and hatched larvae were incubated for 1 h at 95 °C in 100 µL of Base Solution (25 mM NaOH, 0.2 mM EDTA, pH = 12) and shaking. The solution was cooled down on ice, then 100 µL of Neutralization Solution (40 mM Tris–HCl pH = 5.0) was added and vortexed. 2 µL of the total volume was used in a 25µL PCR reaction. To determine the genotypic sex, a pair of primers (for *O. latipes* and H:Y^*lat*^: Dmrt1andbY_Fw03 5′- AGTGCTCCCGCTGCCGGAAC-3′; Dmrt1andbY_Rv02 5′GACTACAAATCCCAAGCTCCT-3′) was used that amplifies fragments of both *dmrt1a* (1145 bp) and *dmrt1bY* (900 bp), yielding one PCR product (*dmrt1a*) in XX genotypes, and two PCR products (*dmrt1a* and *dmrt1bY*) in XY genotypes.

For *O. curvinotus* and hybrids derived (H:Y^*cur*^), a DNA extraction was performed as follows: fins and embryos were collected and homogenized in extraction buffer (0.1 M EDTA, 0.2%SDS, 1 M Tris pH = 8, 200 µg/mL proteinase K). The total genomic DNA was extracted and purified using phenol, chloroform:isoamyl alcohol (24:1) and cold ethanol 100% for precipitation. After a 70% ethanol rinse, the DNA pellet was resuspended in 100 µl TE buffer (10 mM Tris–HCl, 1 mM EDTA, pH = 8). To genotype *O. curvinotus* and H:Y^*cur*^, a pair of primers (FW1_H2 5′-AAGTGGTGGTGAAGAATGA-3′, RV1_H2 5′-AAGTTGTAGTAGGAGGTTTCCA-3′) was used to amplify fragments of both *dmrt1a* (1850 bp) and *dmrt1bY* (1500 bp), yielding one PCR product (*dmrt1a*) in XX genotypes, and two PCR products (*dmrt1a* and *dmrt1bY*) in XY genotypes. The PCR products were resolved on 1% agarose gels.

### RNA extraction and transcriptome sequencing

Two individual ovaries (XX female, XY female), two testes of individual interspecific hybrids (XY male), three individual ovaries and three pools of three testes from wild type *O. latipes*, Carbio strain, and *O. curvinotus*, respectively were homogenized in TRIzol^®^ reagent (Invitrogen, Waltham, Massachusetts, USA). Total RNA was isolated and purified using RNeasy^®^ Mini kit (Qiagen, Venlo, Netherlands) according to the manufacturer’s instructions. RNA quality was assessed by determining the RNA Integrity Number (RIN) using an Agilent 2100 Electrophoresis Bioanalyzer Instrument (Agilent Technologies 2100 Expert). RNA samples with RIN > 8 were used for sequencing.

RNA sequencing libraries were constructed following the standard TruSeq Illumina mRNA library preparation protocol (www.illumina.com; Illumina Inc., BGI, Hong Kong), with a read length of 150 and sequencing depth for paired ends of 62–72 million reads per sample.

### Transcriptome analysis

Transcriptome sequences were mapped to the *O. latipes* reference genome (Ensembl Release 93) using the RNA-sequence aligner STAR with ‘--quantMode GeneCounts’ (https://github.com/alexdobin/STAR/releases). For the analysis of the expression data and the generation of plots different Bioconductor/R packages were used. Differentially expressed genes between testis and ovary were detected by DESeq2 [31] for wild type and mutants. Genes were considered to be differentially expressed, if *p* value ≤ 0.05 AND log2FC ≤ -2 (higher expression in female) and log2FC ≥ + 2 (higher expression in male). Heat maps for genes was considering with log2FC > 2 AND baseMean > 1000 were plotted and genes showing comparable regulation between male and female wild type and hybrids were selected. Functional clustering was made using DAVID Bioinformatics Resources 6.8 (https://david.ncifcrf.gov/). Correspondence analysis was calculated using the R/bioconductor package ade4 (function: dudi.coa) and plotted using the package rgl.

### Sequence analysis

GenBank accession number sequences BAB92012.1 for Dmrt1bY *O. latipes* and BAC65995.1 for Dmrt1bY *O. curvinotus* were used for amino acid alignments and protein structure prediction. The multiple alignment software Molecular Evolutionary Genetics Analysis version 7.0 (MEGA7) for bigger datasets^25^ was employed for alignment amino acid sequences. For protein structure prediction Raptor X (Källberg et al., 2012, http://raptorx.uchicago.edu/) was used.

### Histology

Embryos from different stages (35 to 40) larvae at 5, 10, 20 and 30 days after hatching (dah) were sacrificed. The head was used to genotyping as described above. The trunk of embryos and larvae and gonads from adult fish were dissected and fixed in Karnovski solution (2% glutaraldehyde and 4% paraformaldehyde in Sörensen buffer [0.2 M, pH 7.2]) for 24 h at 4 °C. Then, samples were washed in water, dehydrated in increasing concentrations of ethanol and embedded in Historesin Technovit 7100 (Kulzer, Hanau, Germany). Serial sections of 3 µm thickness were obtained and counterstained with hematoxylin & eosin (HE).

## Results

### Sex reversal and sterility in *O. latipes*/*O. curvinotus* F1 hybrids

Reciprocal crosses were performed between *O. latipes* and *O. curvinotus.* The corresponding hybrids were designated according to the origin of their Y-chromosomes: either hybrid:Y^lat^ (H:Y^lat^) or hybrid:Y^cur^ (H:Y^cur^). Both crosses resulted in similar proportions of XX females, XY males and sex reversed XY females (Table 1). When the parental female was *O. curvinotus* and the parental male *O. latipes* (H:Y^lat^), 100% of the phenotypic females were XX, while 71% of the XY animals were male and 29% developed as females (sex reversal). In the reciprocal cross, 100% of the females were XX, while 76% of the XY were males and 24% were sex reversed (Table 1).

**Table 1:** Genotypic and phenotypic sexes of the hybrids from *O. latipes* (carbio strain) and O. curvinotus (F1 crossings).

Cross (♀ × ♂)	XX		XY		Total n
	Female (%)	Male (%)	Female (%)	Male (%)	
*O. curvinotus* × *O. latipes*	32 (100)	0 (0)	7 (29)	17 (71)	56
*O. latipes* × *O. curvinotus*	41 (100)	0 (0)	10 (24)	31 (76)	82

**Figure 1:**
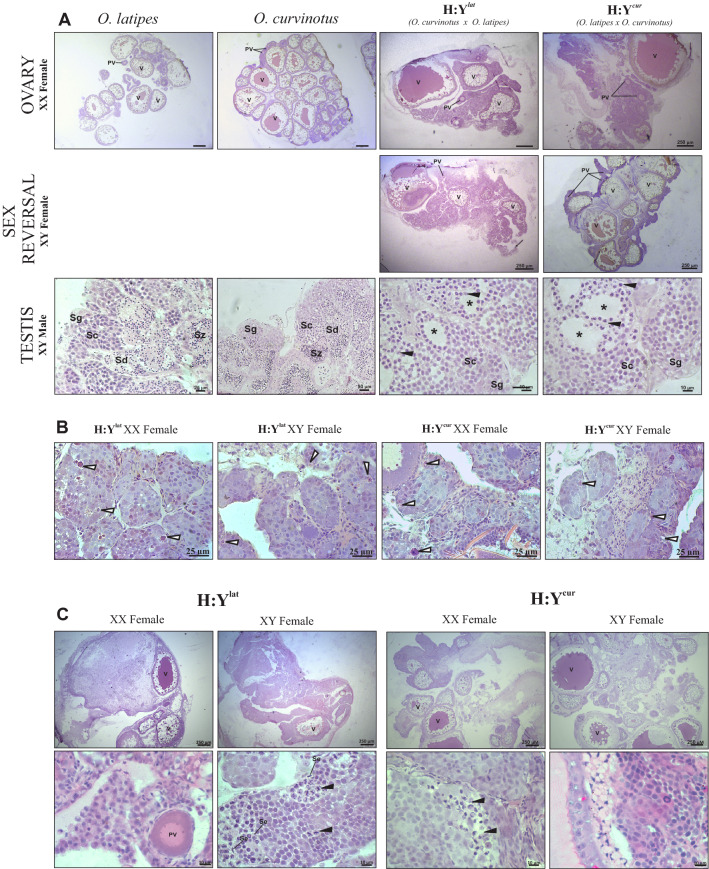
Morphology analysis of adult gonad of parental *O. latipes*, *O. curvinotus* and hybrids. (**A**) Wild type parental fish and hybrids (H:Y^*lat*^ and H:Y^*cur*^) male and female (XX and XY). In the parental ovaries, more vitellogenic oocytes are present in comparison to the hybrids. Hybrid testis present empty seminiferous tubules (star). (**B**) Higher magnification of H:Y^*lat*^ XX female and H:Y^*cur*^ XY male. Ovaries of hybrids show increased numbers of germ cell clusters. (**C**) Gonad histology of H:Y^*lat*^ XX female and XY female with 2.5 years old fish. *V* vitellogenic oocytes, *PV* pre-vitellogenic oocytes, *Se* sertoli cells, *Sg* spermatogonia, *Sc* spermatocyte, *Sd* spermatid, *Sz* spermatozoa, *Black arrow* spermatocyte arrested in meiosis, *empty seminiferous tube, *White arrow* cells in apoptosis.

The ovaries of adult females from both parental species typically consist of large amounts of vitellogenic and some pre-vitellogenic oocytes. In both hybrid offspring, H:Y^lat^ and H:Y^cur^, XX as well as XY-sex reversed females apparently had less vitellogenic oocytes in comparison to the parental females (Fig. 1A). Strikingly, ovaries from hybrids of both genotypes showed many clusters of germ cells as well as apoptotic cells (Fig. 1B). Nevertheless, XX and XY-sex reversed females were fertile.

Testes of parental *O. latipes* and *O. curvinotus* displayed a typical restricted lobular structure in which lobules show an organized progression of developing germ cells, with spermatogonia confined to the distal end of the lobule. In hybrid males, the morphology of testis was similar to that of the parentals, but no sperm was observed within the lobules. (Fig. 1A). In addition, testes of hybrids showed spermatocytes arrested in meiosis, spermatocytes organized in cysts, and spermatogonia (Fig. 1A). Hybrid males did not show reproductive behavior and were not able to stimulate oviposition in parental females.

One year old hybrid females were fertile. Their capacity to reproduce was nevertheless lost in older animals. Analyses of the gonadal morphology of female hybrids older than two years revealed intersex gonads in both XX and XY females, with prominent reduction of pre-vitellogenic and vitellogenic oocytes, and presence of spermatocytes arrested in meiosis II (Fig. 1C). The testicular portion of the intersex gonad could only be detected by histological analyses, and it is located at the germinal epithelium and spread throughout the tissue with no preferential localization.

### Gonad development in parental and hybrid embryos

Because the analysis of the adult hybrids and their gonads already indicated an ontogenetic discrepancy compared to parentals, histological analyses were performed during embryonic and larval development (stage 36 until 30 dah) in order to investigate the complete gonadal sex differentiation (Suppl. Figs. 1 and 2).

In XX embryos of the parental *O. latipes* and *O. curvinotus*, the first sign of meiosis (leptotene/zygotene) was observed around stage 38. Interestingly, in XX hatchlings of *O. latipes* many differentiated pre-vitelogenic oocytes are present at 10 dah, while in *O. curvinotus* gonadal development was much slower (Suppl. Figs. 1 and 2A). By comparing the parental females with the XX hybrids, we observed that the H:Y^lat^ XX fish had cells in meiosis (leptotene/zygotene) as early as stage 35 and the H:Y^cur^ at stage 37, indicating an earlier onset of meiosis as compared to the parental species (Suppl. Fig. 1). However, the ovaries of XX hybrids developed slower and had no pre-vitellogenic oocytes when inspected between 10 and 30 dah, showing an apparent increase in germ cell number at 10 dah when compared to parental fish (Suppl. Fig. 2A).

It is not possible to predict which XY hybrid embryo will develop ovary or testis during the sex determination period. Thus, we compared the development of the gonads from XY embryos only to both XY parentals (Suppl. Fig. 2B). In XY fish, we noted an increase of germ cell numbers in clusters, which apparently exceeded the numbers in parental XY fish at 30 dah. This indicates a difference in the timing of germ cells proliferation between XX and XY hybrids as well as between hybrids and parental gonadal development (Suppl. Fig. 2B).

### Expression analyses of adult gonads of parental *O. latipes* and *O. curvinotus*

To elucidate the molecular basis of the abnormal sexual development in the interspecies hybrids, we performed a transcriptome analysis of parental and hybrid gonads. Considering the overall differential expression profiles of our samples in a principle component analyses (PCA) revealed that differences between the ovaries of parentals *O. latipes* and *O. curvinotus* are higher than the differences between testis. Hybrid testis and ovaries presented an intermediate expression pattern when compared to the parentals (*x*-axis), indicating no general preference to one species over the other (Suppl. Fig. 3). In addition, all hybrid ovaries presented an intermediate expression pattern between ovary and testis, indicating an increase of the male signaling in those individuals (Suppl. Fig. 3).

**Figure 2:**
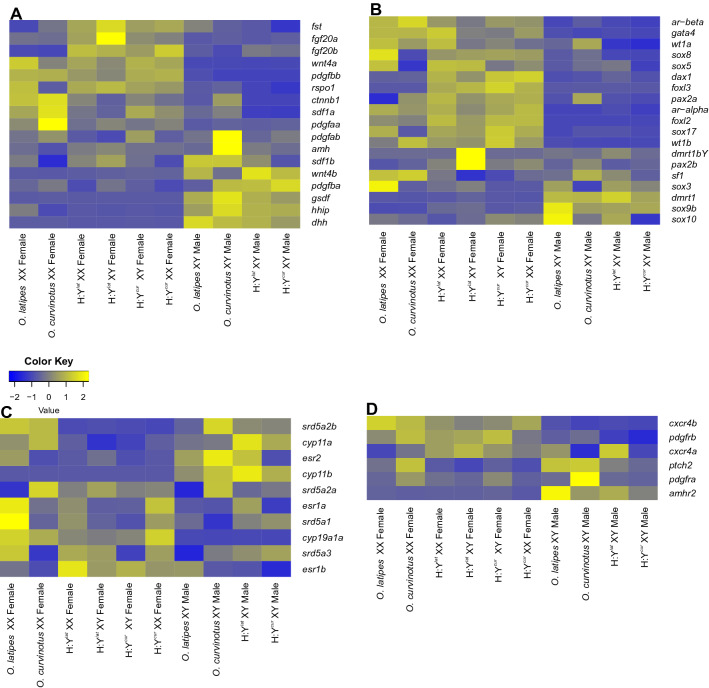
Heatmaps of sex-related genes in male and female adult gonads of parental and hybrids. Growth factors (**A**), transcription factors (**B**), steroidogenic enzymes and receptors (**C**), and growth factor receptors (**D**). Transcripts with higher expression levels are in yellow, while lower expression is in blue.

Despite both species share the same sex-determining gene (*dmrt1bY*), differential expression analysis of adult male and female gonads of parental *O. latipes* and *O. curvinotus* revealed substantial differences. Considering the intraspecific threshold values of LogFC ≥ 2 as higher expression in females, and LogFC ≤ − 2 and *p* value ≤ 0.05 as higher expression in males, only about half of the genes are similarly expressed in both species (Suppl. Fig. 4). For several genes known to be part of the sex determination network, testis of *O. curvinotus* presented higher expression of *amh*, *pdgfra* and *srd5a2a* than testis of *O latipes* males. In females, *pax2a*, *pdgfaa* and *srd5a2a* are higher expressed in *O. curvinotus* than in *O. latipes*, while *rspo1*, *esr1a*, *esr2*, *sox3*, *sox5* and *sox8* have higher expression in *O. latipes* when compared to *O. curvinotus* ovaries (Fig. 2). However, the majority of the sex-related genes showed similar expression levels among the individuals, in particular *dmrt1bY, dmrt1a, gsdf, foxl2, cyp19a1a* (Fig. 2).

### Gonadal expression analyses of hybrids

The gonadal morphologies from male hybrids were similar (Figure 1A), independent of the origin of the Y chromosome. Similarly, the ovaries, with or without Y chromosome, presented a similar structure compared to the parental females. In order to observe the transcriptome alterations in the hybrids, we extracted the equally differentially expressed genes in testes of both male hybrids compared to the parental species, and those of ovaries from all female hybrids (XX and XY) to the parental species.

In H:Y^lat^ and H:Y^cur^ males there were only 4 commonly up-regulated and 47 down-regulated genes. None of these genes has been linked to sperm production so far (Suppl. Fig. 5) (Suppl. Table 1). There were 47 genes exclusively regulated in H:Y^lat^ testis (9 up-regulated, and 38 down-regulated), and 185 genes regulated in H:Y^cur^ testis (55 up-regulated, e.g. *star,* and 130 down-regulated) (Suppl. Fig. 5) (Suppl. Table 2). Interestingly, like in the parentals, *dmrt1bY* is not expressed in adult testis of H:Y^cur^, but it was up-regulated in H:Y^lat^.

**Figure 3:**
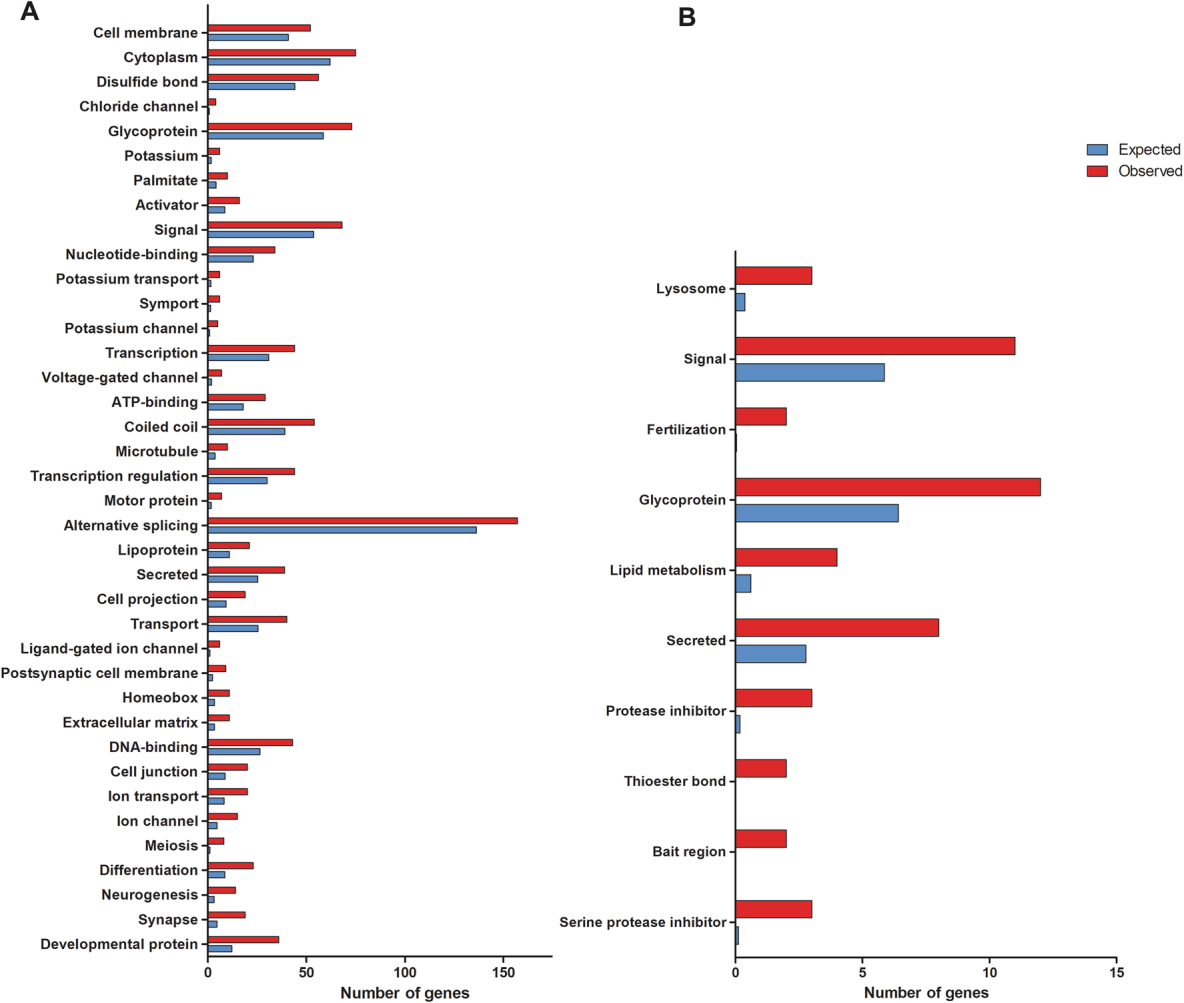
Enriched categories of up-regulated (**A**) and down-regulated (**B**) genes in ovaries of all hybrids. Blue indicating the number of expected genes given the gene selection would be random, red indicating the number of observed genes in the respective gene set. Significance of GO-terms is decreasing from bottom to top. Regulated is defined as *p* value ≤ 0.05 and log2FC ≤ ± 2.

Investigating the genes that are equally expressed in ovaries of all hybrids when compared to both parental species, we observed 401 up-regulated while 75 genes were down-regulated (Suppl. Table 3). Functional category analyses showed that genes related to meiosis and germ cell differentiation (*dazl*, *dmc1*, *scp3*, *ddx4/vasa*, *nanos2*, *pou3f1*, *pou5f2*, *rec8*) were strongly enriched in all hybrids (Fig. 3A) (Suppl. Table 3), which might reflect the increase of germ cell number observed in both XX and XY female hybrids. Important down-regulated genes showed category enrichment in all hybrids for fertility (*zp1* and *zp4*) and ovary development (*bmp15*), in agreement with the reduced ability of the hybrids’ oocytes to be fertilized (Fig. 3B) (Suppl. Table 3). Interestingly, growth and transcription factors known to be important for female gonad development such as *fgf20b*, *dax1* and *foxl3* presented strong up-regulation in ovary of all hybrids (Fig. 2).

Comparing H:Y^lat^ XX with H:Y^cur^ XX females revealed that only about 1% (274 genes) of all transcripts were significantly different. Similarly, in the comparison of H:Y^lat^ XX with H:Y^lat^ XY, we observed around 1% of the transcripts being regulated (277 genes). In addition, about 1,5% (375 genes) of the transcriptome showed up or down-regulation when H:Y^cur^ XX were compared with H:Y^cur^ XY. Interestingly, however, only 9 genes are commonly regulated in H:Y^lat^ XY and H:Y^cur^ XY females, 91 genes are regulated only in H:Y^lat^ XY, and 170 genes are exclusively regulated in H:Y^cur^ XY ovaries (Suppl. Fig. 6) (Suppl. Tables 4 and 5).

**Figure 4:**
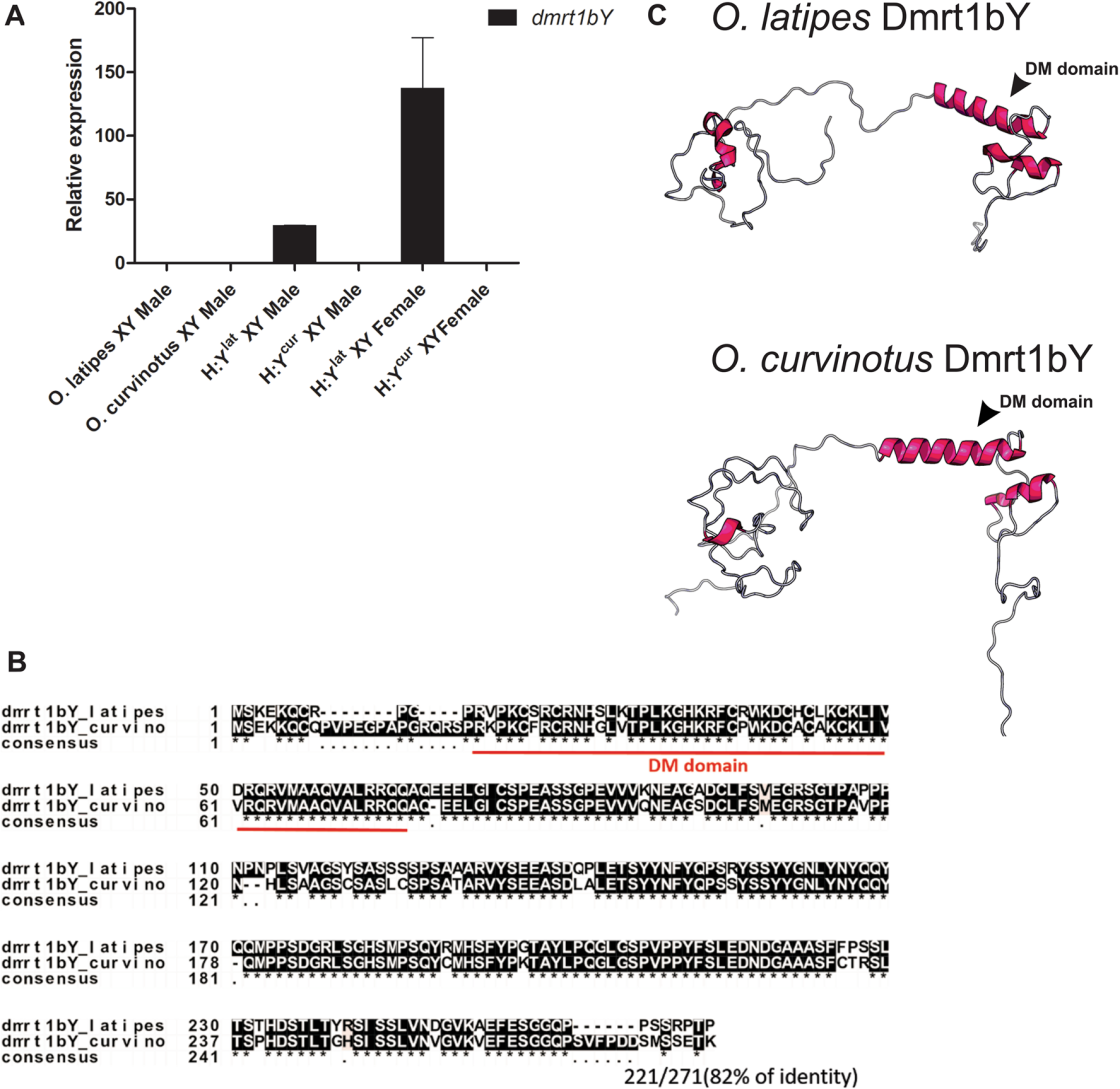
Expression and protein structure analyses of *dmrt1bY*. (**A**) Normalized read counts for *dmrt1bY* transcripts in parental and hybrids that contain the Y chromosome. (**B**) Alignment of predicted amino acid sequence of Dmrt1bY for *O. latipes* and *O. curvinotus*. The red line shows the DM domain. (**C**) Putative protein of Dmrt1bY for *O. latipes* and *O. curvinotus.* The DM domain (in pink) presents the typical zinc-finger-like DNA binding structure.

Remarkably, similar as in H:Y^lat^ XY testis, expression of *dmrt1bY* in H:Y^lat^ XY ovaries was higher than in all other XY hybrids, including also the parental males (14 times higher than *O. latipes* and 30 times higher than *O. curvinotus*) (Fig. 4A). We also checked for qualitative differences between the genes of both species. The Dmrt1bY amino acid sequences from *O. latipes* and *O. curvinotus* have 82% of identity (DM domain 85%) (Fig. 4B). Analysis of Dmrt1bY putative protein structure for both species showed only small differences between them, with the DM domain appearing to have a conserved structure (Fig. 4C).

## Discussion

The *dmrt1bY* gene is a duplicated copy of the autosomal *dmrt1* (*dmrt1a*) gene, which was then inserted into another chromosome thereby generating a proto-Y chromosome in an ancestor of *O. latipes* and *O. curvinotus*^26,27^. Despite having the same sex-determining gene, our results revealed that genes related to sex determination pathway showed some quantitative and qualitative differences in the total mRNA expression profile of the gonads. These differences in the downstream pathways could explain the sex reversal, sterility, and the development of intersex gonads in the hybrids.

The *dmrt1bY* gene was described to act during embryonic development at the critical sex-determining stage, but its expression decreased at later stages remaining lower in the adult gonad^28^. However, XY female and male hybrids showed higher expression of *dmrt1bY* in adult gonads, but only when the Y chromosome comes from *O. latipes*. The expression of *dmrt1bY* only in H:Y^lat^ hybrids suggests a different regulation of *dmrt1bY*^*lat*^ and *dmrt1bY*^*cur*^ alleles. In *O. latipes*, transposons containing cis-regulatory elements invaded the promoter of *dmrt1bY*, critically regulating its expression. The Izanagi element is one of these regulatory sequences which contains multiple binding sites for Sox5 and Pax2 transcription factors, as well as a Dmrt1 binding site^29^. We were not able to identify this element in the promoter region of *dmrt1bY* of *O. curvinotus* (data not shown), suggesting differences in the mechanisms that regulate the expression of *dmrt1bY* in each species. This might explain the observed discrepancy in expression of the orthologs from both species.

Given the high morphological and functional similarity of the sex reversed ovaries in both hybrids, an involvement of *dmrt1bY* expression in the primary processes of sex reversal in H:Y^lat^ hybrids is difficult to assign. It has been shown that mutations leading to lower expression levels of *dmrt1bY* induce XY male-to-female sex reversal in medaka^30^. Expression of *dmrt1bY* in sex-reversed ovaries was also reported in hormone-induced XY male-to-female sex reversal in *O. latipes*^13^. However, for exerting its biological function as a determiner of the undifferentiated gonad anlage to become a testis, *dmrt1bY* has to be expressed early during the sex determination stage and at sufficient levels. Hence, the *dmrt1bY* gene seems to have a very stage-specific role for the sex determination process, and its expression does not influence the gonadal structure and physiology after the gonad once is determined and differentiated.

The cell biological function of *dmrt1bY* in determining sex is to impose a mitotic arrest on the primordial germ cells in the developing gonad at the sex-determination stage^16^. The autosomal *dmrt1a* gene of male medaka is expressed later than *dmrt1bY* and is essential for testis differentiation and maintenance^31^. Our results show that meiosis occurs later in *O. curvinotus* and the embryo development timing is slower compared to *O. latipes*. We suggest that the time at which *dmrt1bY* expression starts is different between species. This will create an asynchrony between *dmrt1bY* expression and the timing of meiosis. The Dmrt1bY imposed arrest on PGC mitosis will not be effective because it would occur too early or too late. Interfering with *dmrt1bY* expression at the sex-determining stage of *O. latipes* (onset of meiosis in female embryos) led to a precocious increase in PGC numbers and to female gonadal development in XY fish^16^. We hypothesize that this is one of the reasons why a certain fraction of XY hybrids do not develop as males but as females. In hybrids between *Mus domesticus* males and B6 (C57BL/6J) females strain, the sex determining gene *Sry* was shown to have delayed expression which leads to sex reversal^32^.

Genes involved in gametogenesis, germ cell differentiation and meiosis were up-regulated in female hybrids, which is reflected in the gonadal morphology showing an increased numbers of germ cells and spermatocytes at meiosis. Notably, in both XX and XY female hybrids at about two years of age, the ovaries developed into ovotestis independent from the presence or absence of the Y chromosome. Strikingly, *foxl3* was strongly up-regulated in all female hybrids compared to parental ovaries. A *foxl3* knockout led to the development of ovotestis in medaka^33^. It was suggested that *foxl3* may play a role in sexual fate decision of germ cells towards female by suppressing spermatogenesis^34^. We suggest that the high expression of *foxl3* in female hybrids is correlated with the increased number of germ cells.

Previous studies revealed that the number of vitellogenic oocytes is considerably lower in hybrids, because most of the oocytes cannot enter into the pachytene stage of prophase I^22^. Our transcriptome analyses associate this observation to the molecular biology of the hybrid ovaries. Genes related to oocyte maturation and fertility were extremely down-regulated in ovaries of hybrids, while the meiotic pathway genes were increased. Of note, with respect to gonad function the adult XY male hybrids from both crossing (H:Y^*lat*^ and H:Y^*cur*^) were more affected than female showing full loss of the capacity to reproduce (sterility), due to the absence of sperm in the gonads, while females were fertile and could produce mature oocytes. Female hybrids between *O. latipes* and *O. curvinotus* were reported to produce diploid eggs, which could be due to the observed reduction of oocyte maturation and fertility gene expressions, probably by a checkpoint control of homologous chromosome-paring^22^. We suggest that the ploidy increase and the higher germ cell number, could lead to an imbalance between the male and female-related genes, and activating the spermatogenesis at late stages.

The recurrent situation noted in our interspecific hybrids is known as hybrid male sterility (HMS), where the male is the infertile sex. Several scenarios and hypotheses have been put forward to explain the lower fitness of interspecific hybrids^35,36^. Sex chromosomes apparently play a central role in the evolution of reproductive incompatibilities between closely related species. Haldane’s rule (HR) states that in interspecific hybrids the heterogametic sex is more likely to exhibit inviability or infertility than the homogametic sex due to a “genetic imbalance”^35,36^. HR applies to the *O. latipes/curvinotus* hybrids, because all XY males were sterile independent of the origin of the Y chromosome. Nevertheless, our data shows that hybrids of both sexes presented negative impacts in fertility, independent from the origin of the sex chromosome and sex-determining gene. Although the males were completely sterile, in agreement with the heterogametic sex explanation, XX and XY female hybrids presented disrupted phenotype and similar transcriptome profiles. Hence, our data adds to HR that the incompatibility effect of being sterile is a combination of the Y chromosome and the development of a male gonad. On the contrary, in the sex reversed XY females, the situation of being a hybrid is not connected to disrupted fertility. To confirm the importance of the heterogametic sex in the *Oryzias* genus in hybrid sterility, similar analyses should be performed between two species containing the ZZ/ZW sex chromosome system (e.g., *Oryzias javanicus* and *Oryzias hubbsi*).

**Figure 5:**
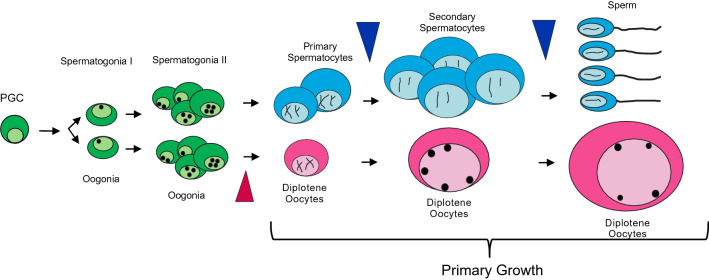
Scheme of the steps of spermatogenesis and oogenesis. Arrowheads indicate the stages that were disrupted in the H:Y^lat^ and H:Y^cur^ hybrids.

HR can be explained by the Bateson–Dobzhansky–Muller model, which states that genetic incompatibilities evolve by fixation of alternative alleles after separation of two genetic lineages at many loci, so that when hybridization occurs, alleles on the X chromosome of one species are incompatible with the interacting genes on the Y chromosome of the other species^37–39^. The results observed in our study resembles the situation widely demonstrated in different vertebrate and invertebrate species, in which hybrid males suffer postmeiotic sterility problems (e.g. sperm bundling and motility) more often than pre-meiotic or meiotic problems^40,41^ (Fig. 5). Further cytogenetic and molecular analyses of meiosis in *Oryzias* hybrids would help to elucidate the exact mechanism of HMS.

## Supplementary Information

Supplementary Figures.

Supplementary Tables.
