# Supplementary material for: Sexual development dysgenesis in interspecific hybrids of Medaka fish
Source: Sci Rep. 2022 Mar 30;12:5408. doi: 10.1038/s41598-022-09314-6 (PMC8967909; doi:10.1038/s41598-022-09314-6)
Supplement: Supplementary file 1 — Supplementary Figures. [file 41598_2022_9314_MOESM1_ESM.pdf]

Supplementary figure 1. Meiosis entry and oocyte development analyses in parental and hybrids.

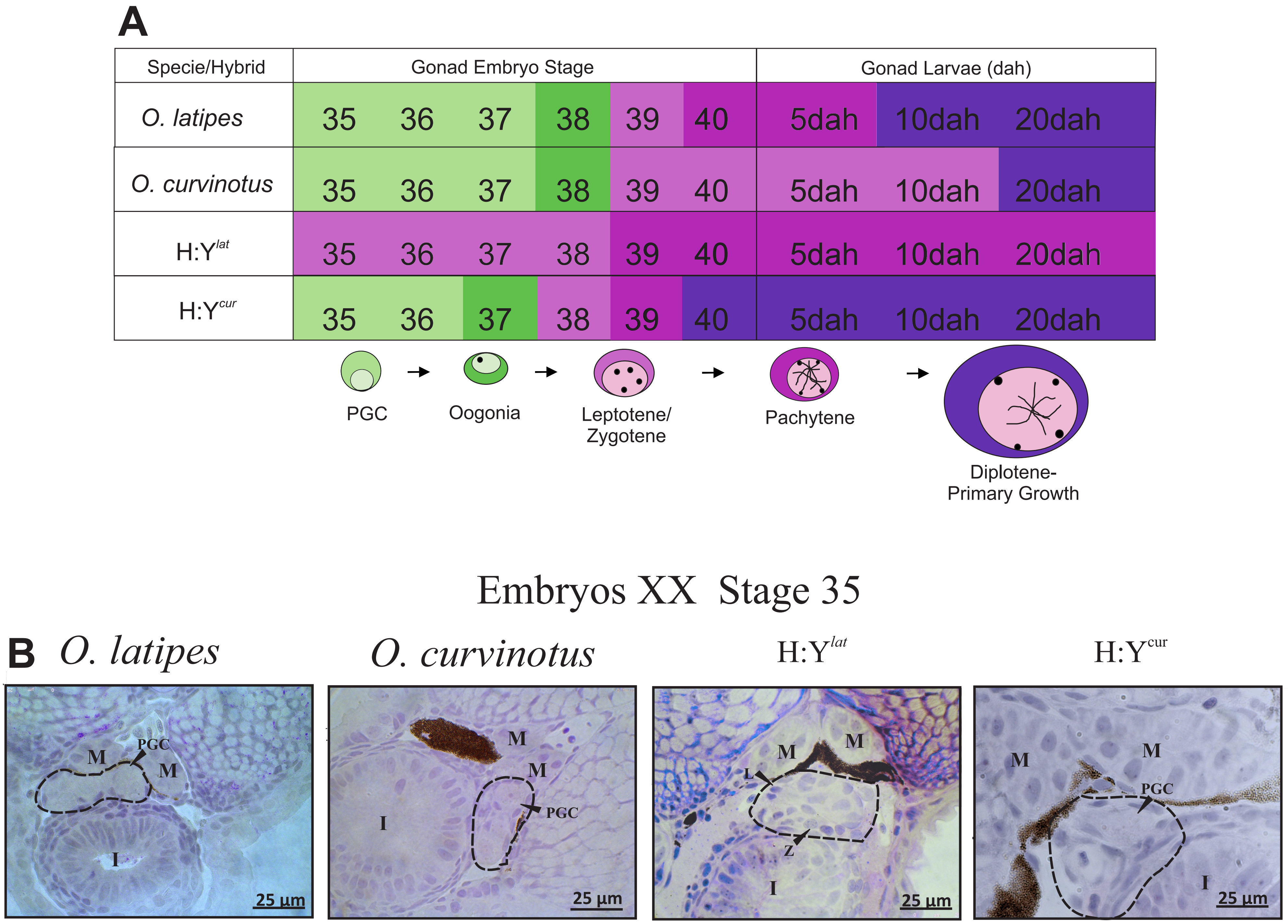

A. Female gonadal development in parental fish and hybrids H:Y^{lat} and H:Y^{cur} from stage 35 to 20 day after hatching (dah). Each square corresponds to an embryo stage. Each color from green to red correspond to different stages of meiosis: PGC: primordial germ cells, oogonia, leptotene/zygotene, pachytene and diplotene/primary growth. **B.** Histology of female embryos at stage 35 in *O. latipes*, *O. curvinotus*, H:Y^{lat} and H:Y^{cur}. M: mesonephros, PGC: Primordial germ cells, I: Intestine, L: Leptotene, Z: Zygotene.

Supplementary figure 2. Histological analysis of the gonad development at 10 dah, 20dah and 30dah in hybrids H:Y*lat*, H:Y*cur* and parental fish. A.

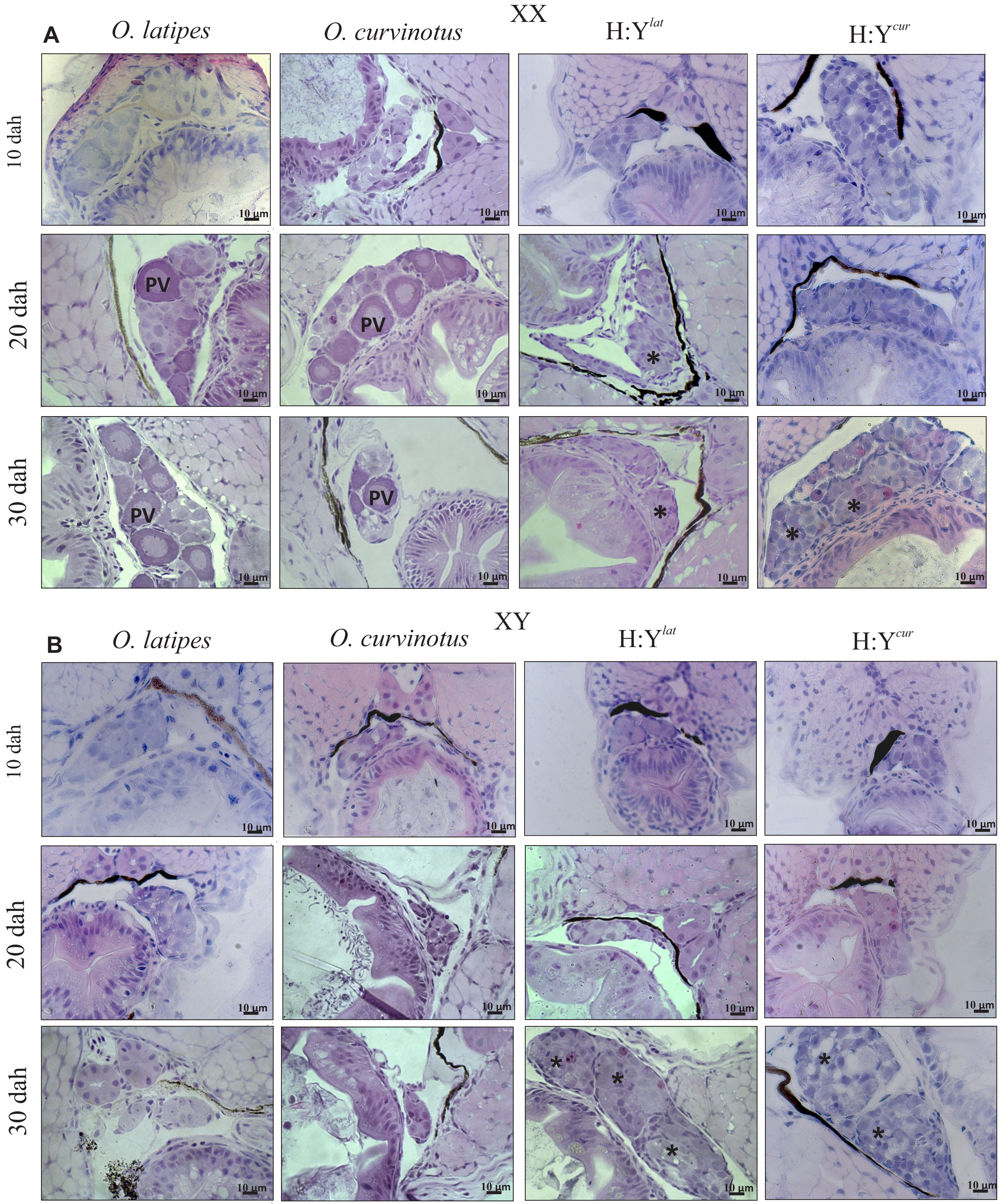

Genotyped larva XX gonads. **B.** Genotyped larva XY gonads. **PV:** pre-vitellogenic oocytes, *: cluster of germ cells.

Supplementary figure 3. 3D plot of the correspondence analysis showing the relationship between male, female, parental and hybrid fish samples.

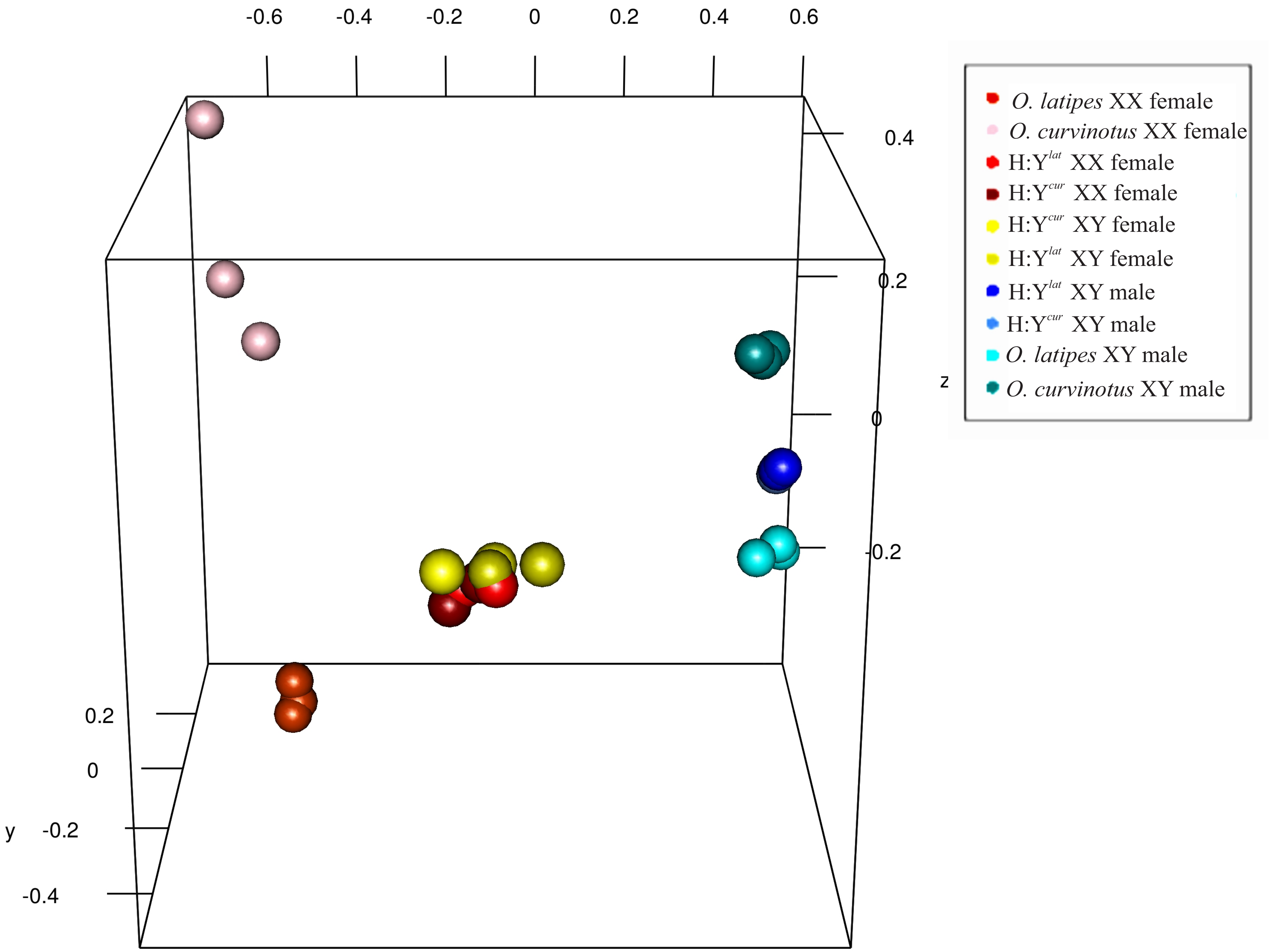

Supplementary figure 4. Venn diagram of male-biased and female-biased gene expression in *O. latipes* and *O. curvinotus* parental fish.

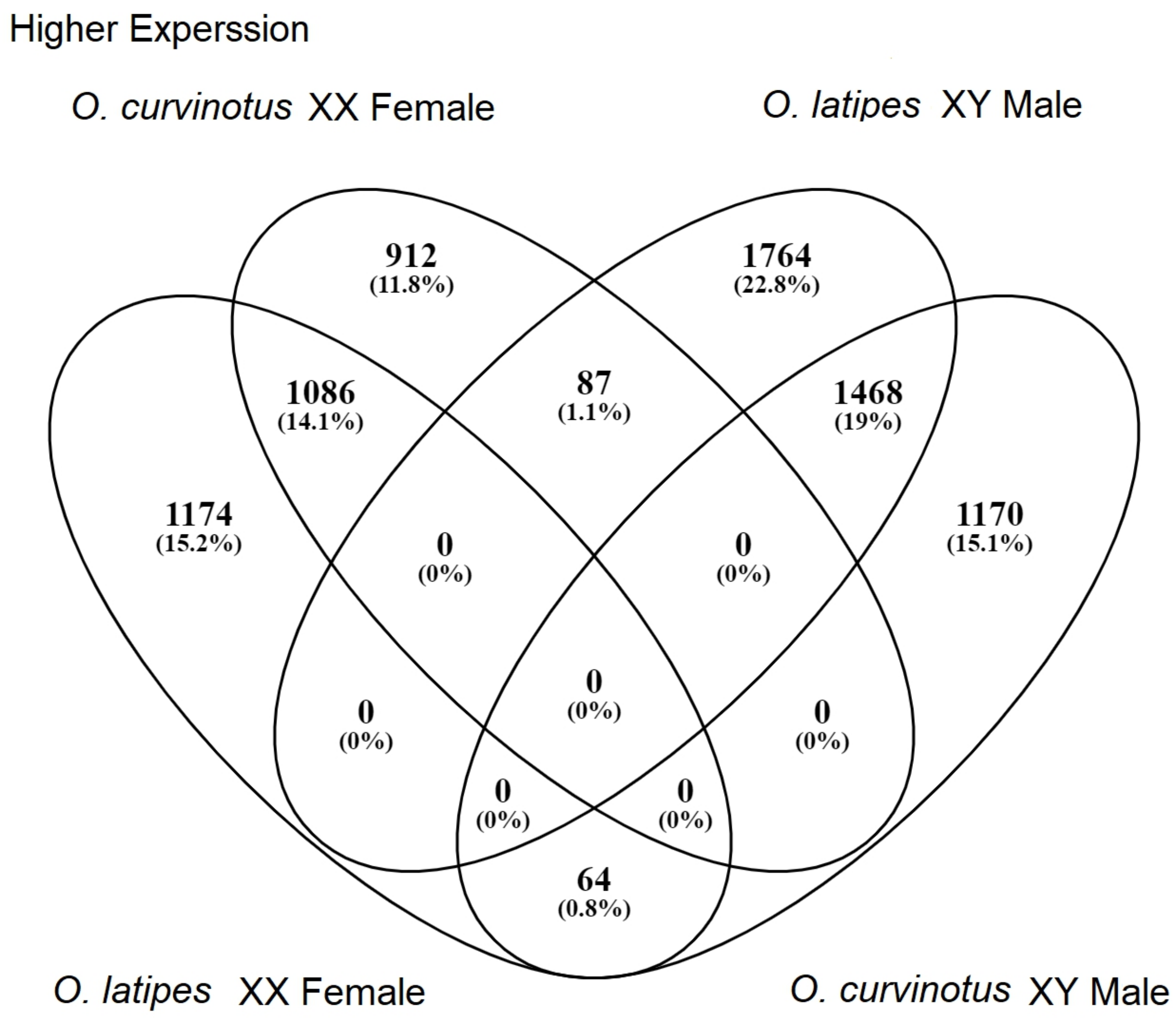

Venn diagram displays the relation between the number of genes that have higher expression for one of the sexes in each species.

Supplementary figure 5. Venn diagram of regulated genes in both H:Y^{lat} and H:Y^{cur} hybrid males in comparison to parental testis of both species.

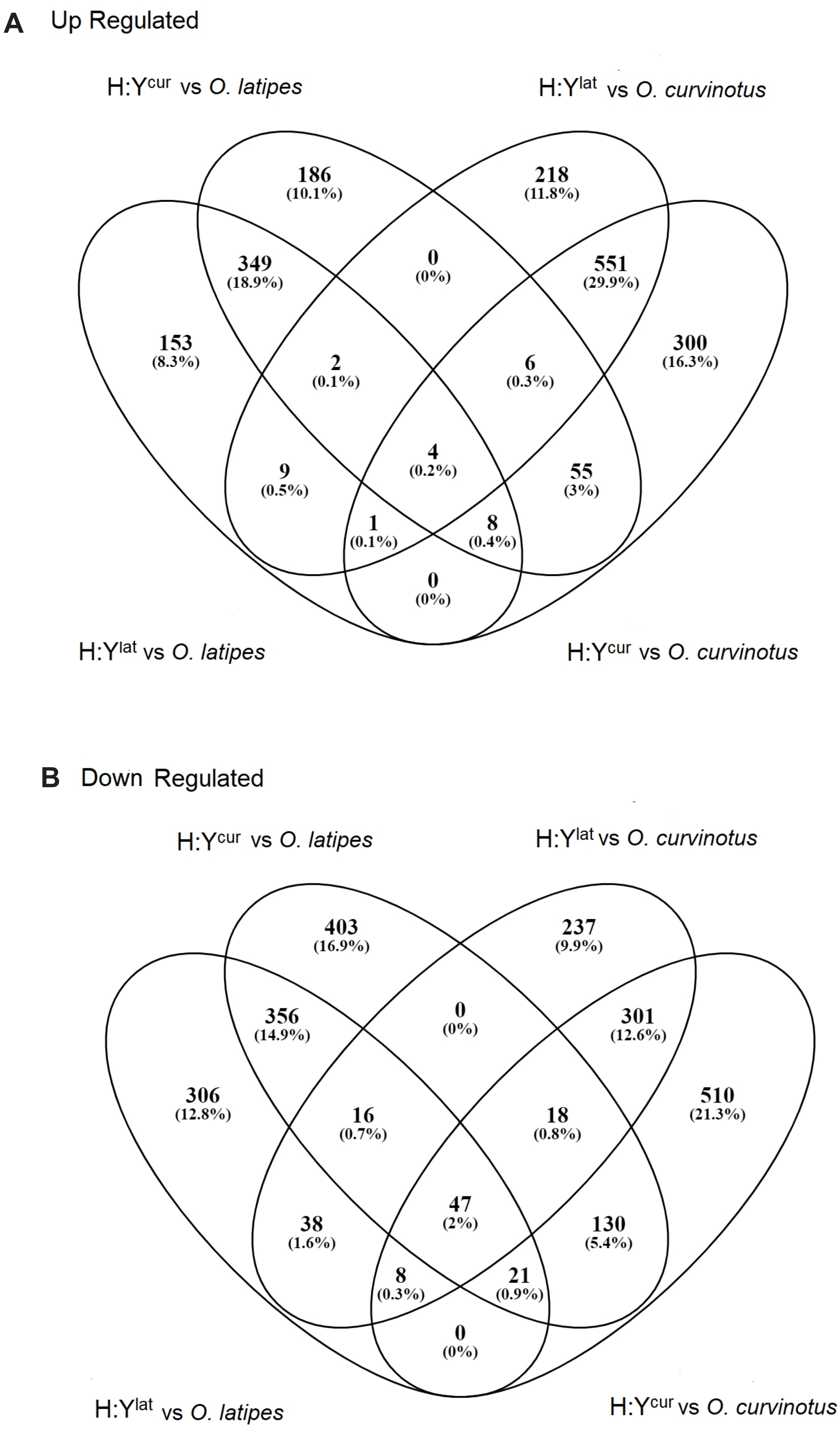

Venn diagrams displays the relation between the number of genes that were up-regulated (**A**) and down-regulated (**B**) in male hybrids when compared to parental males.

Supplementary figure 6. Venn diagram of regulated genes sex reversal XY ovaries in comparison to XX ovaries from both H:Y*lat* and H:Y*cur* hybrids.

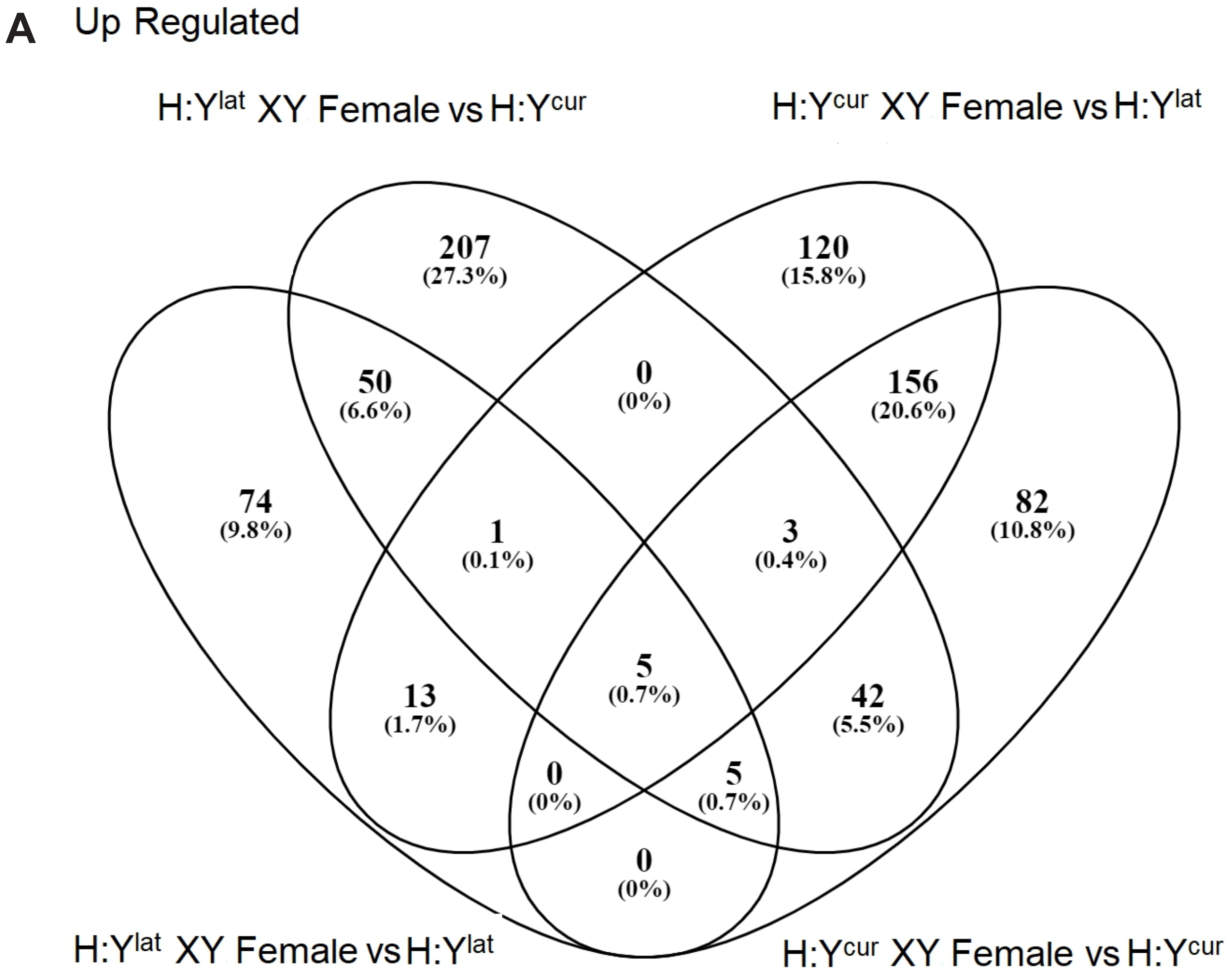

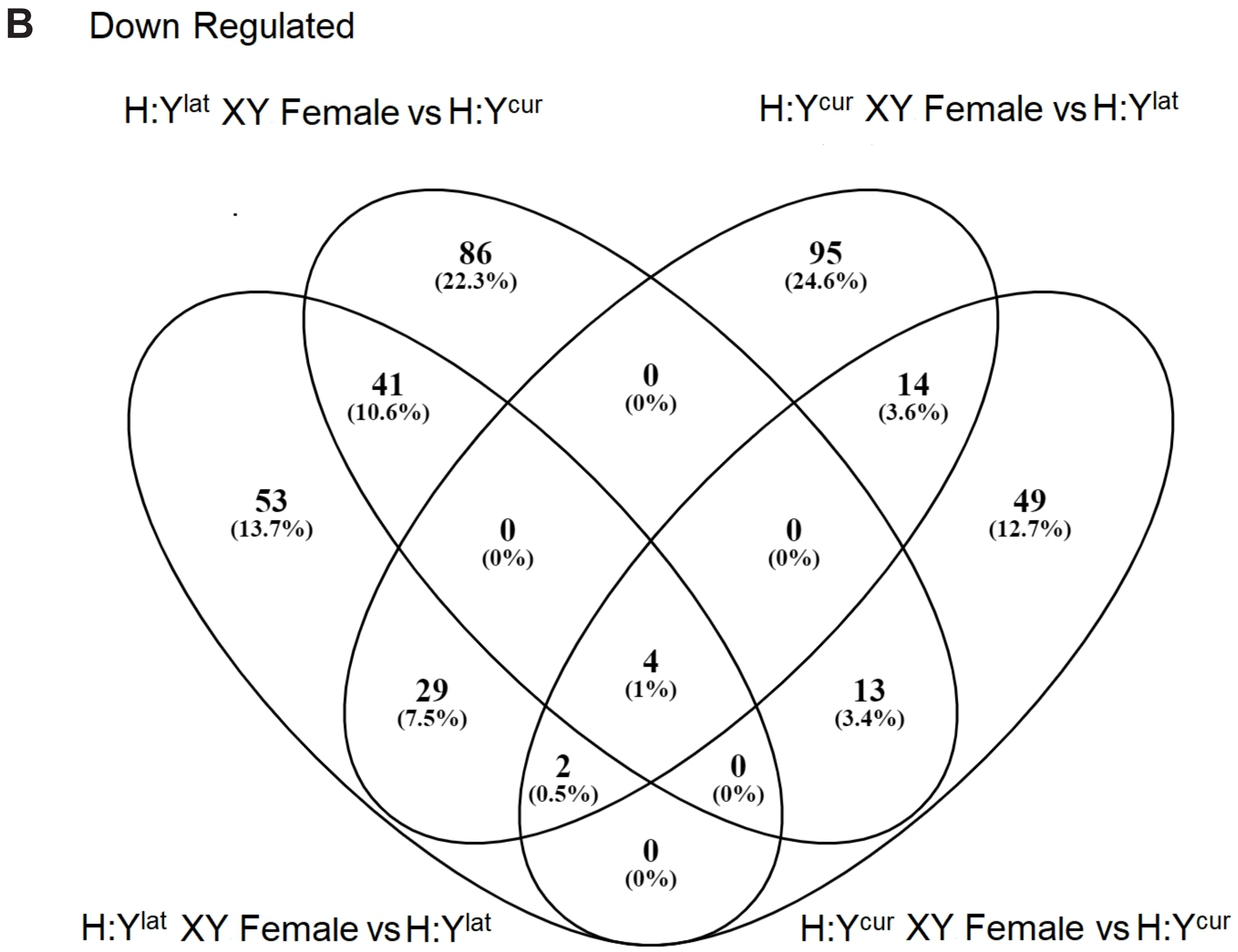

Venn diagrams displays the relation between the number of genes that were up-regulated (**A**) and down-regulated (**B**) in XY female hybrids when compared to XX ovaries from hybrids.
